# Complete genome sequence data of chitin-degrading *Bacillus velezensis* RB.IBE29

**DOI:** 10.1016/j.dib.2023.109815

**Published:** 2023-11-17

**Authors:** Dinh Minh Tran, Thi Huyen Nguyen, Anh Dzung Nguyen

**Affiliations:** Institute of Biotechnology and Environment, Tay Nguyen University, Buon Ma Thuot, Dak Lak 630000, Vietnam

**Keywords:** Complete genome sequence, *Bacillus velezensis*, Genes involved in antifungal activity, Genes involved in plant growth promotion

## Abstract

This work reports the complete genome sequence of chitinolytic *Bacillus velezensis* RB.IBE29 recently isolated from the rhizosphere of black pepper cultivated in the Central Highlands region of Vietnam. This bacterium had strong antagonistic activity against phytopathogens and possessed a novel chitinase system. The complete genome of strain RB.IBE29 was sequenced using the platforms of Illumina (2×150 PE) and Oxford Nanopore technologies. Assembly showed that strain RB.IBE29 has one 3,957,092-bp circular chromosome with 46.5 % G+C content. DFAST analysis revealed the genome contains 3819 protein-coding genes, 27 rRNAs, 86 tRNAs, 1 tmRNA, 144 pseudogenes, and shares an ANI value of 97.51 % with that of reported *B. velezensis* NRRL B-41580. The *B. velezensis* RB.IBE29 genome possesses at least 42 genes concerning heavy metal resistance and plant-growth promotion. CAZymes analysis showed that 103 genes coding for carbohydrate-active enzymes were predicted in the genome, including 41 genes for glycoside hydrolases, 34 genes for glycosyl transferases, 3 genes for polysaccharide lyases, 17 genes for carbohydrate esterases, 6 genes for auxiliary activities, and 2 genes for carbohydrate-binding modules. Of these deduced enzymes, at least 8 probably possess activities against phytopathogens, such as family 18 chitinases, family 16 glucanase, and family 46 chitosanase. AntiSMASH analysis exhibited that 15 biosynthetic gene clusters were found in the genome; among them, 5 show no sequence similarity to known bacterial clusters. The raw sequences in this work were deposited in Mendeley Data. The complete genome sequence of strain RB.IBE29 was submitted to the DDBJ/GenBank/EMBL under accession number AP028932. The obtained data provide insight into the biocontrol ability and plant-growth promotion of *B. velezensis* RB.IBE29. The data are valuable for further explorations concerning crop production and other fields using gene expression approaches.

Specifications Table

**Table:** 

Subject	Microbiology: Applied Microbiology
Specific subject area	Molecular biology
Data format	Raw, Filtered, and Analyzed
Type of data	Figures, Tables
Data collection	The genomic DNA of *B. velezensis* RB.IBE29 cells was isolated using the QIAamp DNA mini kit. Genomic libraries for Illumina whole genome sequencing were prepared using the NEBNext Ultra II DNA Library Prep Kit and then sequenced using a MiniSeq machine. Libraries for Oxford Nanopore Technologies whole genome sequencing were prepared using the Ligation Sequencing Kit and then sequenced using a Flongle machine. Finally, bioinformatic tools were used to analyze data.
Data source location	• Institution: Institute of Biotechnology and Environment, Tay Nguyen University • City/Province/Region: Buon Ma Thuot/Dak Lak/The Central Highlands • Country: Vietnam
Data accessibility	1. Raw sequences Repository name: Mendeley Data Data identification number: doi:10.17632/6xp5yb72cd.2 Direct URL to data: https://data.mendeley.com/datasets/6xp5yb72cd/2 2. Genome sequence Repository name: DDBJ/GenBank/EMBL Data identification number: AP028932 Direct URL to data: https://www.ncbi.nlm.nih.gov/nuccore/AP028932

## 1 Value of the Data

• Data provide an insight into the genomics of chitin-degrading *B. velezensis* RB.IBE29 isolated from the Central Highlands of Vietnam.
• Data can be helpful for comparing the genomics of *B. velezensis* originating from the Central Highlands and others.
• Data can be useful for further explorations involved in agricultural cultivation and other fields using DNA recombinant techniques.

## 2 Background

Vietnam is the largest producer and exporter of black pepper in the world. Among the country's regions, the Central Highlands is the capital of black pepper production [1]. *B. velezensis* RB.IBE29 is a chitinolytic bacterium recently isolated from the rhizospheric soil of black pepper grown in the Central Highlands. *In vitro* and greenhouse experiments demonstrated that strain RB.IBE29 possesses high chitin-degrading activity and significantly inhibits the mycelial growth of *Phytophthora*. Field experiments revealed that using *B. velezensis* RB.IBE29 was a good solution for sustainable black pepper production in this region [2], [3]. Strain RB.IBE29 also possesses a novel chitinase system with two family 18 chitinases and an AA10 protein. These chitinases have been expressed in *Escherichia coli* cells and purified. Purified recombinant chitinases possessed high activities against the spore germination of fungi and the egg-hatching of plant-parasitic nematodes [4], [5]. However, the complete genome of this bacterium has yet to be sequenced. This work aimed to sequence and analyze the whole genome of *B. velezensis* RB.IBE29 to establish a dataset and elucidate its biocontrol ability and plant-growth promotion, as well as open the subsequent studies involved in crop production and related fields.

## 3 Data Description

**Fig. 1:**
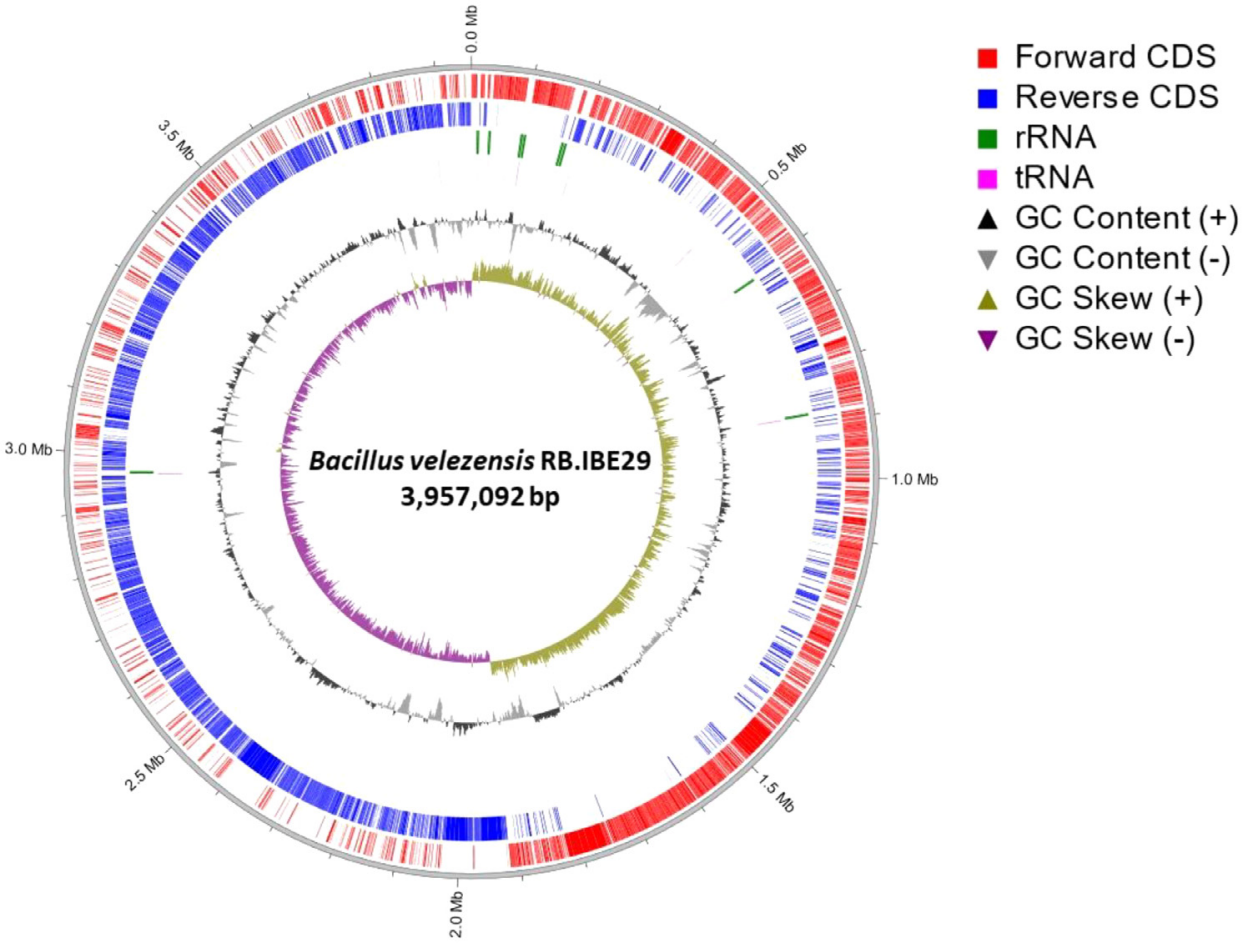
The chromosome of *Bacillus velezensis* RB.IBE29. *Note:* The chromosome was generated using the Proksee server (https://proksee.ca/).

The *B. velezensis* RB.IBE29 complete genome contains 3,957,092 base pairs (bp) with 46.5 % G+C (Fig. 1). The *B. velezensis* RB.IBE29 genome has an ANI value of 97.51 % with the *B. velezensis* NRRL B-41580 genome (GCA_001461825.1). It harbours 3819 protein-coding genes, 27 rRNA genes, 86 tRNA genes, 1 tmRNA gene, and 144 pseudo genes (Table 1). The raw sequences were deposited in Mendeley Data and can be accessed at https://data.mendeley.com/datasets/6xp5yb72cd/2. The genome sequence has been submitted in the DDBJ/GenBank/EMBL under accession AP028932 and can be accessed at https://www.ncbi.nlm.nih.gov/nuccore/AP028932.

**Table 1:** Genome features of *Bacillus velezensis* RB.IBE29.

Features	*B. velezensis* RB.IBE29
No. of contigs	1
Total length (bp)	3,957,092
G+C content (%)	46.5
N50	3,957,092
L50	1
Protein-coding sequences	3819
Hypothetical proteins	530
Functional proteins	3289
rRNA	27
tRNA	86
tmRNA	1
Pseudo genes	144

*Note:* The complete genome features of *B. velezensis* RB.IBE29 were analyzed using the DFAST.

Analysis revealed that 42 genes concerning heavy metal resistance and plant growth promotion were identified from the *B. velezensis* RB.IBE29 genome. Among them, 5 genes related to heavy metal resistance, one gene to inorganic phosphate solubilization, 3 genes to inorganic zinc solubilization, 3 genes to inorganic potassium solubilization, 10 genes to Indole-3-acetic acid biosynthesis, 6 genes to nitrate transport and reduction, 5 genes to ACC biosynthesis, and 9 genes to iron uptake and siderophore production (Table 2).

**Table 2:** Heavy metal and plant growth-promoting genes found in the *Bacillus velezensis* RB.IBE29 genome.

Function	Gene product	Locus
Heavy metal resistance		
Cd^2+^, Co^2+^, Zn^2+^	Cadmium, cobalt, and zinc/H(+)-K(+) antiporter	RBIBE_05270
Cd^2+^, Co^2+^, Zn^2+^	Cadmium, zinc, and cobalt-transporting ATPase	RBIBE_30860
As^3+^, As^5+^	Arsenical pump membrane protein	RBIBE_33510
As^3+^, As^5+^	Arsenical pump membrane protein	RBIBE_35880
Cr^6+^	Chromate transport protein	RBIBE_33610
Inorganic phosphate solubilization	4-hydroxy-3-methylbut-2-enyl diphosphate reductase	RBIBE_23780
Inorganic zinc solubilization	Zinc-dependent sulfurtransferase SufU	RBIBE_29960
	Zinc-specific metallo-regulatory protein	RBIBE_23720
	Zinc-transporting ATPase	RBIBE_13600
Inorganic potassium solubilization	Ktr system potassium uptake protein A	RBIBE_14240
	Ktr system potassium uptake protein A	RBIBE_28250
	Ktr system potassium uptake protein B	RBIBE_13270
Indole-3-acetic acid biosynthesis	Tryptophan synthase alpha chain	RBIBE_21120
	Tryptophan synthase beta chain	RBIBE_21130
	Tryptophan-tRNA ligase	RBIBE_10890
	Anthranilate phosphoribosyltransferase	RBIBE_21160
	Anthranilate synthase component 1	RBIBE_21170
	Aminodeoxychorismate/anthranilate synthase component 2	RBIBE_00760
	Indole-3-glycerol phosphate synthase	RBIBE_21150
	Chorismate mutase	RBIBE_21180
	3-dehydroquinate synthase	RBIBE_21190
	Chorismate synthase	RBIBE_21200
Nitrate transport and reduction	Nitrate reductase-like protein NarX	RBIBE_34660
	Nitrate reductase 1 alpha chain	RBIBE_34690
	Nitrate reductase 1 beta chain	RBIBE_34680
	Nitrate transporter NarK	RBIBE_34730
	NADPH-nitrite reductase	RBIBE_02980
	Nitrite reductase small subunit NirD	RBIBE_02970
ACC biosynthesis	Acyl-CoA dehydrogenase	RBIBE_22770
	Acyl-CoA dehydrogenase	RBIBE_18460
	Acyl-CoA dehydrogenase FadE	RBIBE_30110
	Acyl-CoA dehydrogenase family protein	RBIBE_04320
	Acyl-CoA synthetase	RBIBE_09970
Iron uptake and siderophore production	Iron ABC transporter permease	RBIBE_01700
	Iron ABC transporter permease	RBIBE_35840
	Iron ABC transporter permease FeuC	RBIBE_01690
	Iron chelate uptake ABC transporter family permease subunit	RBIBE_30600
	Iron ABC transporter permease	RBIBE_35850
	Iron ABC transporter substrate-binding protein FeuA	RBIBE_01710
	Iron-hydroxamate ABC transporter substrate-binding protein	RBIBE_35860
	Siderophore ABC transporter ATP-binding protein	RBIBE_30240
	Siderophore ABC transporter ATP-binding protein	RBIBE_30590

*Note:* The heavy metal and plant growth-promoting genes in the *B. velezensis* RB.IBE29 complete genome were analyzed using the DFAST.

In this work, 103 carbohydrate-active enzymes were deduced from the *B. velezensis* RB.IBE29 genome (Table 3). Of the deduced enzymes, there were 41 glycoside hydrolases, 34 glycosyltransferases, 3 polysaccharide lyases, 17 carbohydrate esterases, 6 auxiliary activities, and 2 carbohydrate-binding modules. Of those, 4 enzymes probably possess antifungal activity against phytopathogens, including one family 16 beta-glucanase, two family 18 chitinases, and one family 46 chitosanase. In addition, 4 genes encoding peptidases (loci RBIBE_22550, RBIBE_36110, RBIBE_13610, and RBIBE_27440), which possibly exhibit activity against the growth of plant pathogens, were identified from the genome of *B. velezensis* RB.IBE29.

**Table 3:** CAZymes predicted in the *Bacillus velezensis* RB.IBE29 genome.

Class	Family	Number of sequences
Glycoside hydrolases	1	4
	3	1
	4	3
	5	1
	11	1
	13	4
	16	1
	18	2
	23	2
	26	1
	30	2
	32	3
	43	4
	46	1
	51	2
	53	1
	68	1
	73	2
	109	1
	126	1
	171	1
	177	1
	179	1
Glycosyltransferase	1	3
	2	16
	4	6
	8	1
	26	1
	28	2
	51	4
	83	1
Polysaccharide lyases	1	2
	9	1
Carbohydrate esterases	1	3
	4	7
	6	1
	7	1
	9	1
	12	1
	14	3
Auxiliary activities	1	1
	4	1
	6	1
	7	2
	10	1
Carbohydrate-binding modules	50	2

*Note:* The CAZymes in the *B. velezensis* RB.IBE29 complete genome were analyzed using the dbCAN2 metaserver.

**Fig. 2:**
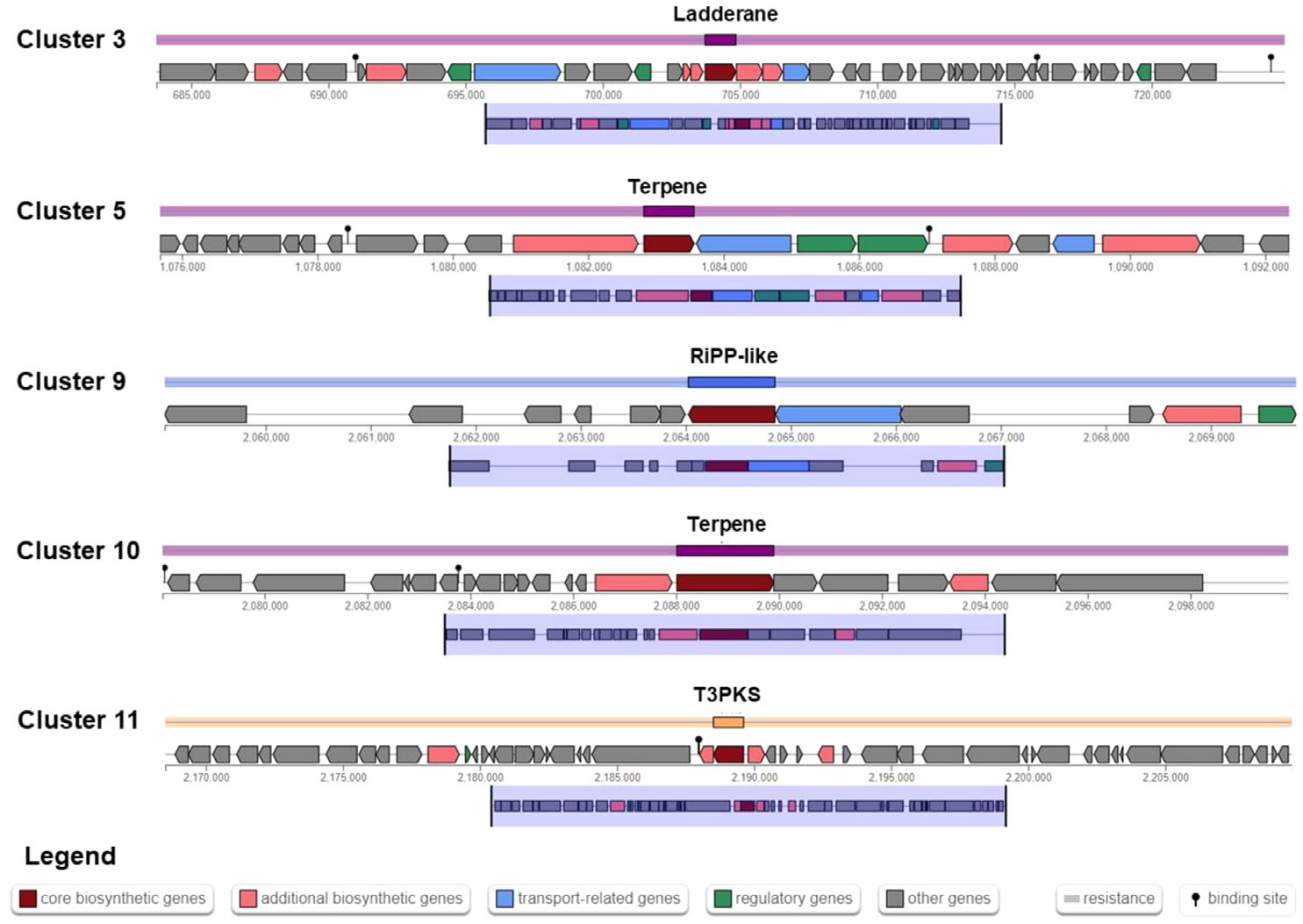
Novel biosynthetic gene clusters identified from the *Bacillus velezensis* RB.IBE29 genome. *Note:* Biosynthetic gene clusters in the *B. velezensis* RB.IBE29 complete genome were analyzed using the AntiSMASH.

AntiSMASH analysis showed that the genome of *B. velezensis* RB.IBE29 harbors 14 putative gene clusters responsible for antimicrobial metabolite biosynthesis. Interestingly, 5 clusters show no sequence similarity to known bacterial gene clusters (Table 4, Fig. 2).

**Table 4:** Biosynthetic gene clusters found in the *Bacillus velezensis* RB.IBE29 genome.

Cluster	Position (nucleotide to nucleotide)	Metabolite type	Most similar known cluster	Identity (%)
Cluster 1	98,145…119,475	Lanthipeptide-class-ii	Loseolamycin A1/loseolamycin A2	8
Cluster 2	311,542…376,351	NRPS	Surfactin	82
Cluster 3	683,729…724,850	Ladderane	-	-
Cluster 4	949,553…990,797	PKS-like	Butirosin A/butirosin B	7
Cluster 5	1,075,676…1,092,351	Terpene	-	-
Cluster 6	1,418,712…1,505,085	TransAT-PKS	Macrolactin H	100
Cluster 7	1,732,690…1,832,616	TransAT-PKS/T3PKS/NRPS	Bacillaene	100
Cluster 8	1,905,702…2,042,434	NRPS/transAT-PKS/betalactone	Fengycin	100
Cluster 9	2,059,038…2,069,813	RiPP-like	–	–
Cluster 10	2,078,017…2,099,900	Terpene	–	–
Cluster 11	2,168,518…2,209,618	T3PKS	–	–
Cluster 12	2,337,494…2,431,261	TransAT-PKS	Difficidin	100
Cluster 13	3,041,788…3,093,581	NRP-metallophore/NRPS/RiPP-like	Bacillibactin	100
Cluster 14	3,606,970…3,648,388	Other	Bacilysin	100
Cluster 15	3,793,712…3,816,900	Lanthipeptide-class-ii	Mersacidin	100

*Note:* Biosynthetic gene clusters in the *B. velezensis* RB.IBE29 complete genome were predicted using the AntiSMASH.

## 3 Experimental Design, Materials and Methods

Genomic DNA extraction of *B. velezensis* RB.IBE29 cells, Illumina library preparation, and sequencing were performed as described by Tran et al. [6]. Libraries for Oxford Nanopore Technologies (ONT) whole genome sequencing were prepared using the Ligation Sequencing Kit (Oxford Nanopore Technologies, USA), following the manufacturer's protocol. The library was then sequenced using a Flongle machine (Oxford Nanopore Technologies, UK) [7].

*De novo* assembly, annotation, and bioinformatic analysis of data were done as previously described [6], [7]. Briefly, Fastp v.0.23.1 [8] was used to analyze raw sequence reads. Unicycler v.0.4.8 [9] and pipeline flye-medaka-polca [10], [11] were used to *de novo* assemble the analyzed reads. Finally, the web-based annotation pipeline, DFAST [12], was used to annotate the complete genome sequence of *B. velezensis* RB.IBE29. An average nucleotide identity analysis (ANI) was analyzed as described previously [13] to determine the genetic relationship of strain RB.IBE29 and known bacteria based on the genome sequence. The carbohydrate-active enzymes database, dbCAN2 metaserver [14], was used to analyze proteins related to carbon metabolism. The antiSMASH v.6.0 [15] was used to predict gene clusters responsible for antimicrobial metabolite biosynthesis.

## Limitations

Not applicable.

## Ethics Statement

The current work does not involve human subjects, animal experiments, or any data collected from social media platforms.

## CRediT authorship contribution statement

**Dinh Minh Tran:** Conceptualization, Methodology, Investigation, Formal analysis, Software, Data curation, Validation, Visualization, Writing – review & editing. **Thi Huyen Nguyen:** Investigation, Formal analysis, Software. **Anh Dzung Nguyen:** Data curation, Validation, Visualization.

## Data Availability

Complete genome sequence of Bacillus velezensis RB.IBE29, the chitinolytic bacterium isolated from the rhizospheric soil of black pepper (Original data) (Mendeley Data) Bacillus velezensis RB.IBE29 DNA, complete genome (Original data) (NCBI)
